# Associations of diet, physical activity and polycystic ovary syndrome in the Coronary Artery Risk Development in Young Adults Women’s Study

**DOI:** 10.1186/s12889-020-10028-5

**Published:** 2021-01-06

**Authors:** Annie W. Lin, David Siscovick, Barbara Sternfeld, Pamela Schreiner, Cora E. Lewis, Erica T. Wang, Sharon S. Merkin, Melissa Wellons, Lyn Steffen, Ronit Calderon-Margalit, Patricia A. Cassano, Marla E. Lujan

**Affiliations:** 1 Department of Nutrition, Benedictine University, 5700 College Road, Lisle, IL 60532 USA; 2 Department of Preventive Medicine, Northwestern University, 680 N Lake Shore Drive, Suite 1400, Chicago, IL 60611 USA; 3 New York Academy of Medicine, 1216 Fifth Avenue, New York, NY 10029 USA; 4 Division of Research, Kaiser Permanente Northern California, 2000 Broadway, Oakland, CA 94612 USA; 5 Division of Epidemiology and Community Health, University of Minnesota, 1300 S. 2nd St., Suite 300, Minneapolis, MN 55454 USA; 6 Division of Epidemiology, University of Alabama at Birmingham, 210C Ryals Public Health Building, 1720 2nd Ave S, Birmingham, AL 35294-0022 USA; 7 Obstetrics and Gynecology, Cedars-Sinai, 444 S. San Vicente Ave, Suite 1002, Los Angeles, CA 90048 USA; 8 Division of Geriatrics, University of California Los Angeles, 10945 Le Conte Avenue, Suite 2339, Los Angeles, CA 90095 USA; 9 Division of Diabetes, Endocrinology, and Metabolism, Vanderbilt University, 1215 21st Avenue South, Medical Center East, South Tower, Suite 8210, Nashville, TN 37232 USA; 10 Division of Epidemiology and Community Health, University of Minnesota, 300 West Bank Office Building, Minneapolis, MN 55454 USA; 11 Hadassah-Hebrew University Braun School of Public Health. Hadassah Medical Center, PO Box 12272, 91120 Jerusalem, Israel; 12 Division of Nutritional Sciences, Cornell University, Savage Hall, Ithaca, NY 14853 USA

**Keywords:** Nutrient, Physical activity, Polycystic ovary syndrome, Oligomenorrhea, Hyperandrogenism

## Abstract

**Background:**

Current evidence supports the adoption of healthy diet and physical activity (PA) behaviors in patients with polycystic ovary syndrome (PCOS), given the positive effects of those behaviors on physical well-being. An improved understanding of the associations between diet and PA with PCOS is needed to ascertain whether tailored dietary and PA recommendations are needed for this population. Thus, we investigated the associations of diet and PA with PCOS and its isolated features.

**Methods:**

Cross-sectional study. Of the 748 women who were included in this study from the Coronary Artery Risk Development in Young Adults (CARDIA) Women’s Study, 40 were classified as having PCOS, 104 had isolated hyperandrogenism (HA) and 75 had isolated oligomenorrhea (OA). Dietary intake was measured using the CARDIA diet history questionnaire and diet quality was scored using the Alternative Healthy Eating Index 2010; a higher score indicated a better quality diet. Self-reported PA was measured using a validated interviewer-administered questionnaire. Polytomous logistic regression analyses examined the associations between diet and PA with PCOS, HA, and OA status (outcomes), adjusting for age, race, total energy intake, education, and/or body mass index. The threshold for statistical significance was set at *p* < 0.05.

**Results:**

Mean age of the participants was 25.4 years (SD 3.6) and 46.8% of participants were Black women. There was little to no association of total energy intake, nutrients, diet quality, and PA with PCOS, HA or OA status.

**Conclusion:**

Energy intake, nutrient composition, diet quality, and PA were not associated with PCOS, supporting recent PCOS guidelines of using national recommendations for the general population to encourage health-promoting behaviors among women with PCOS. However, longitudinal studies evaluating changes in diet and physical activity in relation to the development and/or the progression of PCOS are needed to establish a causal association.

**Supplementary Information:**

The online version contains supplementary material available at 10.1186/s12889-020-10028-5.

## Background

Polycystic ovary syndrome (PCOS) is a complex endocrine condition traditionally characterized by ovulatory dysfunction and androgen excess [1]. In addition to reproductive complications, women with PCOS often present with overweight or obesity and associated obesity-related complications, such as insulin resistance, impaired glucoregulatory status, and cardiometabolic dysfunction [2]. Rates of overweight and obesity in PCOS (up to 88% of patients) well exceed that seen in the general population and suggest that obesity can either contribute to PCOS development and/or is a consequence of PCOS pathogenesis [2]. Accordingly, expert recommendations support modifications in diet and physical activity primarily targeted at weight loss or management as a means to improve health outcomes in PCOS [2–5]. Current diet and physical activity recommendations for women with PCOS reflect those for the general population or other clinical populations (e.g., diabetes mellitus) that do not necessarily account for the unique biology and/or psychosocial variables associated with PCOS [6, 7]. An understanding of associations between dietary intake and physical activity levels with PCOS is ultimately needed to determine the relevance of tailored therapies for this patient population.

We previously noted inconsistent findings related to differences in energy intake and dietary composition between women with and without PCOS [8]. Approximately half of the 10 studies reviewed reported differences in total energy [9–13] and fat intake [9, 10, 13–15] in women with PCOS versus a reference group. Three studies did not detect any association(s) between diet and PCOS [16–18]. With respect to physical activity levels, only two studies reported longer sitting intervals in women with PCOS than the reference [11, 14]. Most showed no differences in overall activity between groups [9, 11–14, 18]. Collectively, published studies do not provide definitive evidence on associations of diet, physical activity and PCOS [8].

Comparisons among previous studies of dietary intake and physical activity levels in relation to PCOS are limited by differences between studies in how PCOS phenotypes were defined [8]. Depending on the PCOS diagnostic criteria used, there are four distinct phenotypes. The National Institutes of Health (NIH) criteria define PCOS by the combined presence of oligomenorrhea (OA) and hyperandrogenism (HA) [1] to identify a relatively homogenous patient population with the most severe risk for reproductive and metabolic dysfunction [5]. The Rotterdam criteria define PCOS by the combined presence of two out of three cardinal features: OA, HA, and polycystic ovarian morphology [19]; the Rotterdam criteria captures a more heterogeneous patient population, including mild PCOS phenotypes (i.e., women with regular menstrual cycles and HA or women with OA and normal androgens) [20]. Milder phenotype(s) may reflect different etiologies and lower risks for metabolic and reproductive sequelae [5]. Differences in metabolic and reproductive risk across PCOS phenotypes may be attributed to the presence of isolated OA versus HA and/or the additive effects of these two features in conjunction with adiposity. Higher adiposity (an outcome closely linked with diet and physical activity) has been reported to be more prevalent in severe (i.e., those with HA) versus mild PCOS phenotypes [20]. There is an emerging consensus that studies should use a more homogenous definition of PCOS [21] or account for phenotypes in their analyses. Studies of usual dietary intake and physical activity levels that consider isolated versus combined cardinal features of PCOS while accounting for body mass index (BMI) are also needed.

While race influences the likelihood of adverse metabolic outcomes in women with PCOS [22–27], few prior studies investigated race as an effect modifier. To our knowledge, prior studies of the association of dietary intake or physical activity levels with PCOS do not investigate differences by race. Previous studies conducted in the United States (US) reported a higher odds and/or prevalence of obesity, hypertension [24, 25], and elevated fasting glucose concentrations in Black versus White patients [23, 26]. Although it is unclear whether race influences the etiology or pathogenesis of PCOS, food choice and medical experiences can vary by race and contribute to differential metabolic manifestations [28]. New recommendations from the international evidence-based guideline for the assessment and management of PCOS acknowledge this knowledge gap and endorse the need for further research on the impact of race/ethnicity in PCOS and the identification of best approaches for treatment across races and clinical phenotypes [29]. To that end, the primary objective of the present study was to investigate the cross-sectional associations of usual dietary intake and physical activity levels with (a) PCOS and (b) its isolated features (i.e. OA or HA alone) as defined by NIH criteria to include a more homogenous patient population. We hypothesized that high energy and fat intake, and high sedentary behavior, were associated with PCOS and its isolated features. We also explored whether race had a modifying effect on these associations as our secondary objective.

## Methods

### Study design and sample

The research questions were addressed using data from the Coronary Artery Risk Development in Young Adults (CARDIA) multi-center longitudinal prospective cohort study. From 1985 to 1986, CARDIA investigators enrolled 5115 Black and White men and women **(Supplemental Figure** **1****)**, ages 18 to 30 years of age, residing in one of four cities: Birmingham, Alabama; Chicago, Illinois; Minneapolis, Minnesota; and Oakland, California [30]. Participants visited research centers at years 2, 5, 7, 10, 15, 20, 25 and 30 after the initial examination (year 0), with a 71% retention rate of the surviving cohort at year 30. The CARDIA study was approved by the institutional review boards of the CARDIA coordinating center and the four participating field centers, and written informed consent was obtained from participants at all examinations. At year 16 (2002–2003), a subset of women was enrolled in an ancillary study (CARDIA Women’s Study; CWS) that investigated associations among androgens, polycystic ovaries and cardiovascular risk factors. Women were eligible if they had participated in the year 15 examination, had at least one ovary and were not pregnant. Eighty-six percent (*n* = 1163) of eligible women in the CARDIA cohort enrolled in the CWS (ages 34–46 years at enrollment to CWS). CWS participants completed questionnaires that retrospectively inquired about their reproductive health history and the occurrence of unwanted hair growth during the ages of 20s and 30s, an age range that for most of the participants coincided with the diet, physical activity, biochemical, and anthropometric data collected during years 0 and 2 of the CARDIA study. We excluded women who: had missing data on PCOS features (*n* = 294), were pregnant at year 2 (*n* = 103), and/or had implausible total energy (kcal) intake (defined as < 600 and > 6000 kcal) (*n* = 18). Thus, the analytic sample comprised 748 women (64% of the CWS cohort). The current study was deemed exempt by the Cornell University IRB since data was de-identified.

### Group definitions

Participants were classified into four mutually exclusive groups: 1) PCOS; 2) isolated hyperandrogenism (HA); 3) isolated oligomenorrhea (OA); and 4) neither PCOS, HA, or OA. NIH criteria [both oligomenorrhea (irregular menstrual cycles) and hyperandrogenism (clinical and/or biochemical)] were used to identify women with PCOS. Oligomenorrhea was defined as self-reported menstrual cycle lengths ≥34 days during the woman’s 20s and 30s (retrospective data collected on reproductive history questionnaire at year 16). Hyperandrogenism was defined as self-reported unwanted hair growth at two or more regions of the body (with the exception of the lower leg and/or underarm) during the woman’s 20s and 30s (retrospective data collected on reproductive history questionnaire at year 16) or biochemical hyperandrogenemia based on assays in year 2 serum samples. Biochemical hyperandrogenemia was defined as total testosterone (T) ≥ 76 ng/dL and/or free T ≥ 0.69 ng/dL. Cut-off points were defined by the 95th percentile of androgen concentrations in CWS women with regular menstrual cycles (20 to 30 days) and no symptoms of unwanted hair growth during their 20s or 30s (*N* = 415). Androgens and sex hormone binding globulin were assayed by the OB/GYN Research and Diagnostic Laboratory at the University of Alabama, Birmingham as previously described [31]. Women who had only one of the two PCOS criteria were classified as either HA or OA.

### Data collection

#### Diet and physical activity measurements

Year 0 dietary data was used, given that the data was collected using the interviewer-administered CARDIA Diet History and was also in close proximity to the year 2 androgen concentrations [32]. Participants reported their dietary intake in the past 28 days, including the frequency and amount of food consumption and methods of food preparation. Forty-six food and beverage subgroups were defined by the Nutrient Data Software for Research (NDSR; University of Minnesota, Minneapolis, Minnesota). The intake of each food or beverage subgroup was calculated by summing the number of daily servings of items included in the subgroup.

Diet quality was scored using the Alternate Healthy Eating Index 2010 (AHEI-2010). The AHEI-2010 includes 11 dietary components (seven food groups, four nutrient groups) [33]. Each dietary component score ranged from zero to ten, with a higher score representing a healthier diet. The total AHEI-2010 score was calculated as the sum of all 11 components and ranged from 0 to 110. The 46 NDSR food and beverage subgroups were sorted into the seven AHEI-2010 food groups. Higher intakes of vegetables, fruits, whole grains, nuts/legumes, long chain omega 3 fatty acids (eicosapentaenoic and docosahexaenoic acids; EPA + DHA), polyunsaturated fatty acids (PUFAs without EPA + DHA) were assigned higher scores. Sugar-sweetened beverages and fruit juices, red and processed meats, *trans*-fats and sodium were assigned scores on a reverse scale due to their association with adverse health outcomes (thus, for these sub-scores a higher score indicates lower consumption). Given the complex and non-linear relation of alcohol to health outcomes, women who consumed 0.5 to 1.5 drinks/day were assigned the highest score (score of 10), women who consumed > 1.5 drinks/day were assigned the lowest scores, and non-drinkers were assigned a score of 2.5 [33].

Self-reported physical activity data were collected during year 0 with the CARDIA Physical Activity History questionnaire, which asked about the frequency of 13 types of moderate and vigorous intensity activities in the past year (e.g., jogging or running, recreational sports, home maintenance) [34, 35]. Physical activity scores were calculated by multiplying the frequency and intensity of each activity and summing across all activities. Additional details on the calculation can be found in previous CARDIA publications [34]. Activity scores were expressed as ‘exercise units’ (EU) since the questionnaire did not ask specifically about the duration of each activity. A score of approximately 300 EU is approximately equivalent to moderate-to-vigorous exercise five times per week for 30 min [34].

#### Sociodemographic and clinical measurements

Sociodemographic data (age, race, education) were collected at year 0 using interviewer-administered questionnaires. For anthropometry data collection at year 0, participants changed into light clothing and removed their shoes prior to anthropometric measurements taken by trained research staff. Weight was obtained using a digital scale (Detecto model 439; Webb City, Missouri) and measured to the nearest 0.2 pounds. Height was collected using a vertical mounted ruler and measured to the nearest 0.5 cm. BMI was calculated as weight in kilograms divided by height in meters, squared.

### Statistical analyses

Statistical analyses were completed using SAS 9.4 (SAS Institute, Cary North Carolina). The statistical significance threshold was set at *p* < 0.05 for the bivariate (i.e., t-test and χ2 test) and logistic regression analyses to compare each of the PCOS, HA, and OA groups with the reference group for the overall sample. Polytomous logistic regression estimated the association of dietary and physical activity (continuous) variables with the odds of PCOS, HA and OA after adjusting for age, race, total energy intake and education in partial models, and extended to include BMI in the fully-adjusted models. To investigate whether associations with PCOS, HA and OA varied by race, regression models examined the interaction(s) between race and the dietary and physical activity variables using covariates from the fully adjusted models. The significance threshold for the effect modification of race on logistic regression models was set at *p* < 0.10 and we did not adjust for multiple testing for the overall analyses given the exploratory nature of these aims to investigate whether diet and physical activity are linked with features of PCOS [36]. Interpretation focused on effect sizes and confidence intervals (CI) to address the precision of the estimates [37]. These results were reproduced by the Cornell Institute for Social and Economic Research.

## Results

### Participant characteristics

Among the 748 participants included in this analysis, 40 (5.3%) women were classified as PCOS, 104 (13.9%) as HA, 75 (10.0%) as OA, and 529 comprised the reference group. Menstrual cycle status, unwanted hair growth and androgen levels differed across the four groups by definition. By contrast, mean age, BMI, diet, and physical activity were similar across groups (Tables 1 and 2). Compared to the reference group (i.e., no PCOS, HA, or OA), there were lower proportions of Black women in the PCOS and OA groups, and a higher proportion of women with advanced degrees in the PCOS group (*p* < 0.05).

**Table 1:** Characteristics of participants in the CARDIA Women’s Study cohort who were included in a study examining the associations between diet and physical activity with reproductive status^a b^

Variable	PCOS (***n*** = 40)	HA (***n*** = 104)	OA (***n*** = 75)	Reference (***n*** = 529)
Age, years	24.7 ± 3.6	25.6 ± 3.8	25.4 ± 3.8	25.4 ± 3.6
Black, n (%)	11 (27.5) ^3^	57 (54.8)	26 (34.7) ^3^	256 (48.4)
Center, n (%)				
Birmingham, Alabama	8 (20.0)	22 (21.6)	14 (18.7)	125 (23.9)
Chicago, Illinois	5 (12.5)	27 (26.5)	18 (24.0)	117 (22.4)
Minneapolis, Minnesota	10 (25.0)	28 (27.5)	15 (20.0)	125 (23.9)
Oakland, California	17 (42.5)	25 (24.5)	28 (37.3)	156 (29.8)
Education, n (%)				
High School or Lower	8 (20.0) ^3^	40 (38.5)	29 (38.7)	177 (33.5)
College	21 (52.5)	55 (52.9)	39 (52.0)	312 (59.0)
Advanced Degree	11 (27.5)	9 (8.7)	7 (9.3)	40 (7.6)
BMI (kg/m^2^)	25.5 ± 5.9	26.3 ± 6.8^3^	25.0 ± 5.8	24.6 ± 5.7
Unwanted Hair, n (%)	33 (82.5) ^3^	60 (57.7) ^3^	0 (0.0)	0 (0.0)
Menstrual Cycle ≥34 Days, n (%)	40 (100.0) ^3^	0 (0.0)	75 (100.0) ^3^	0 (0.0)
Total Testosterone, ng/dL	78.4 ± 65.3^3^	79.9 ± 101.9^3^	35.9 ± 17.5	34.1 ± 17.7
Free Testosterone, ng/dL	0.7 ± 0.6^3^	0.7 ± 1.2^3^	0.3 ± 0.2	0.2 ± 0.2

^a^ Data are expressed as mean (SD) or N (% within each group). *BMI* Body Mass Index, *CARDIA* Coronary Artery Risk Development in Young Adults, *HA* Isolated Hyperandrogenism (elevated testosterone and/or hirsutism at 2 sites or more), *OA* Isolated Oligomenorrhea (≥34 days in menstrual cycle), *PCOS* Polycystic Ovary Syndrome (hyperandrogenism and oligomenorrhea); Reference (neither PCOS nor HA nor OA)

^b^ Statistical Tests: Bivariate analyses using the t-test (continuous variables) or χ2 test (categorical variables)

^3^ *p* ≤ 0.05 (each group vs. reference group)

**Table 2:** Diet and physical activity for all women in the CARDIA Women’s Study cohort by group^a^

Variable	PCOS (*n* = 40)	HA (***n*** = 104)	OA (***n*** = 75)	Reference (*n* = 529)
**Nutrient**				
Energy (kcal /d)	2229.3 ± 879.4	2312.6 ± 981.1	2170.3 ± 925.7	2246.7 ± 912.3
Total Carbohydrate (g/d)	260.0 ± 108.2	270.7 ± 122.6	258.5 ± 114.9	262.5 ± 111.0
Fiber (g/d)	4.9 ± 2.3	5.2 ± 3.0	5.1 ± 3.3	5.0 ± 2.8
Total Protein (g/d)	83.3 ± 29.7	84.7 ± 36.8	78.1 ± 32.4	82.7 ± 35.8
Total Fat (g/d)	93.3 ± 43.2	97.1 ± 46.7	90.4 ± 45.9	94.2 ± 44.7
Cholesterol (mg/d)	357.2 ± 178.8	357.7 ± 165.7	325.2 ± 159.2	354.2 ± 207.4
Total SFA^f^ (g/d)	35.3 ± 18.0	35.5 ± 17.0	33.6 ± 18.3	35.4 ± 17.7
Total MUFA^g^ (g/d)	34.6 ± 16.8	35.8 ± 18.7	32.6 ± 16.9	34.5 ± 17.3
Total PUFA^h^ (g/d)	16.6 ± 7.3	18.8 ± 10.3	17.8 ± 12.1	17.5 ± 9.0
Omega 3 (mg/day)	77.3 ± 93.2	96.3 ± 125.1	77.9 ± 78.3	83.4 ± 129.4
Vitamin A (IU^i^/d)	10,450.2 ± 8330.0	10,453.8 ± 8623.8	11,464.6 ± 12,917.5	10,612.7 ± 11,420.3
Vitamin C (mg/d)	231.6 ± 373.2	292.8 ± 386.7	273.7 ± 452.9	249.1 ± 445.1
Vitamin D (mcg/d)	7.0 ± 4.1	8.5 ± 6.9	7.0 ± 4.9	7.4 ± 6.7
Alpha Tocopherol Equivalents (mg/d)	21.1 ± 62.3	21.0 ± 55.2	11.6 ± 9.2	16.6 ± 44.1
Sodium (mg/d)	3412.6 ± 1561.3	3383.1 ± 1472.6	3265.2 ± 1518.3	3298.8 ± 1530.7
Calcium (mg/d)	1098.3 ± 520.0	1151.1 ± 830.3	1030.4 ± 509.8	1084.4 ± 673.1
Phosphorus (mg/d)	1454.2 ± 500.8	1474.2 ± 637.9	1403.3 ± 584.5	1459.7 ± 629.7
Thiamin (mg/d)	2.0 ± 0.9	2.7 ± 2.8	2.0 ± 0.9	2.4 ± 2.4
Potassium (mg/d)	3075.7 ± 1148.9	3195.1 ± 1577.9	3048.2 ± 1276.9	3120.1 ± 1361.8
Riboflavin (mg/d)	2.5 ± 1.0	3.1 ± 2.9	2.4 ± 1.1	2.8 ± 2.5
Niacin (mg/d)	24.5 ± 8.9	31.2 ± 24.2	24.2 ± 11.0	27.7 ± 23.5
Iron (mg/d)	20.0 ± 9.2	21.8 ± 16.6	19.0 ± 10.0	21.7 ± 33.0
Copper (mg/d)	2.5 ± 1.4	3.0 ± 2.0	2.8 ± 1.8	2.7 ± 1.9
Magnesium (mg/d)	332.2 ± 168.9	356.5 ± 260.8	308.8 ± 126.3	324.2 ± 149.8
Zinc (mg/d)	14.5 ± 6.3	18.2 ± 12.7	16.4 ± 8.9	16.2 ± 11.7
Folic Acid (mcg/d)	565.9 ± 1248.4	507.9 ± 773.2	385.6 ± 275.9	470.7 ± 1922.4
Caffeine (mg/d)	263.1 ± 289.4	279.2 ± 452.2	231.9 ± 320.6	268.2 ± 585.1
**AHEI-2010**				
Vegetables Score	5.0 ± 2.7	5.0 ± 2.7	5.2 ± 2.8	5.0 ± 2.8
Fruits Score	3.5 ± 2.2	3.7 ± 2.9	3.5 ± 2.7	3.5 ± 2.5
Whole Grains Score	3.3 ± 2.0	2.8 ± 2.1	2.9 ± 2.1	3.1 ± 2.3
SSB^j^, Fruit Juice Score	2.3 ± 3.1	1.7 ± 2.9	1.9 ± 2.9	1.7 ± 2.9
Nuts and Legumes Score	4.7 ± 3.4	4.5 ± 3.5	4.1 ± 3.3	4.6 ± 3.5
Red, Processed Meats Score	4.6 ± 3.1	4.7 ± 3.4	5.2 ± 3.3	5.0 ± 3.1
*trans*-Fat Score	3.8 ± 0.9	3.8 ± 1.0	3.8 ± 1.2	3.9 ± 1.1
Long Chain Omega 3 Fats Score	3.0 ± 2.6	3.5 ± 3.0	3.2 ± 2.9	3.1 ± 2.6
PUFA Score	5.8 ± 1.6	6.2 ± 2.1	6.1 ± 2.2	5.9 ± 2.1
Sodium Score	5.6 ± 3.0	5.6 ± 3.0	5.9 ± 3.1	5.8 ± 3.0
Alcohol Score	6.3 ± 2.9	6.2 ± 2.7	5.7 ± 2.8	5.8 ± 3.0
Total AHEI-2010 Score	47.3 ± 11.6	47.8 ± 12.0	47.5 ± 11.7	47.2 ± 11.6
**Physical Activity**				
Moderate Exercise Units	137.8 ± 101.9	126.2 ± 100.9	124.9 ± 99.5	126.3 ± 98.5
Heavy Exercise Units	243.6 ± 175.2	233.8 ± 198.8	252.9 ± 262.6	218.2 ± 191.3
Total Exercise Units	381.4 ± 217.4	360.0 ± 267.2	377.8 ± 324.8	344.5 ± 257.0

^a^ Data are expressed as mean (SD). *CARDIA* Coronary Artery Risk Development in Young Adults, *HA* Isolated Hyperandrogenism (elevated testosterone and/or hirsutism at 2 sites or more), *OA* Isolated Oligomenorrhea (≥34 days in menstrual cycle), *IU*, International Units; Reference (neither PCOS nor HA nor OA), *MUFA* Monounsaturated Fatty Acid, *PCOS* Polycystic Ovary Syndrome (hyperandrogenism and oligomenorrhea), *PUFA*, Polyunsaturated Fatty Acid, *SFA*, Saturated Fatty Acid, *SSB* Sugar-sweetened Beverages

### Association of Dietary Intake and Physical Activity with PCOS and Isolated Features of PCOS

No statistically significant associations were found in models testing individual macro- or micronutrients intake, diet quality, nor physical activity with the odds of PCOS (Supplemental Tables 1 and 2). Further, we found no statistically significant associations of dietary intake and physical activity with isolated features of PCOS. In further analyses of the AHEI-2010 scores, we used a categorical approach and compared the highest and lowest total diet quality scores on their association with PCOS and its isolated features and found no significant associations. We also tested the association of the 11 subscores of the AHEI-2010 with the 3 PCOS phenotypes by race to explore whether there was evidence of race-specific associations. The AHEI-2010 vegetable intake score was inversely associated with the odds of PCOS in Black, but not White women [Table 3; β = − 0.37 (SE 0.20); P_interaction_ = 0.07]. Thus, a one-unit higher vegetable intake score (i.e., an additional ½ serving/day*)* was associated with 31% lower odds of PCOS in Black participants compared to a 45% higher odds of PCOS in white participants. Differential associations by race were also found for whole grain intake scores [Table 3; β_interaction_ = 0.29 (SE 0.16); P_interaction_ = 0.07]. A one-unit higher whole grain intake score, which is ¼ serving/day, was associated with higher odds of PCOS in Black women [OR 1.34 (90% CI: 0.98, 1.84)], but lower odds of PCOS in White women [OR 0.75 (90% CI: 0.54, 1.02)]. Similar to the findings for PCOS, a ¼ serving/day higher whole grain intake was associated with higher odds of OA in Black women, but lower odds of OA in White women [β_interaction_ = 0.22 (SE 0.12); P_interaction_ = 0.07]. Further, a lower sugar-sweetened beverage and fruit juice score (i.e. approximately a ½ serving/week) was associated with higher odds of OA in Black women only [β_interaction_ = 0.15 (SE 0.09); P_interaction_ = 0.09]. Race interactions were not statistically significant for nutrient intake and physical activity with both isolated and combined features of PCOS.

**Table 3:** Summary of statistically significant race-diet interactions in polytomous logistic regression models estimating the associations of food groups with reproductive status^1,2^

AHEI-2010 Subcomponent Score	All Women^3^		Black		White	
	***β***_***interaction***_ ***(SE)***	***P***_***interaction***_	Odds Ratio	90% CI	Odds Ratio	90% CI
**Vegetables**						
PCOS	−0.37 (0.20)	0.07	0.69	0.46, 1.03	1.45	0.98, 2.17
**Whole Grains**						
PCOS	0.29 (0.16)	0.07	1.34	0.98, 1.84	0.75	0.54, 1.02
OA	0.22 (0.12)	0.07	1.25	0.98, 1.58	0.80	0.63, 1.02
**Sugar-sweetened Beverages, Fruit Juice**						
OA	0.15 (0.09)	0.09	1.17	0.97, 1.40	0.86	0.72, 1.03

^1^ AHEI-2010, Alternative Healthy Eating Index 2010; *OA* Isolated Oligomenorrhea (≥34 days in menstrual cycle), *PCOS* Polycystic Ovary Syndrome (hyperandrogenism and oligomenorrhea)

^2^ Model adjusted for covariates: age, total energy intake, education, BMI. Interaction term: diet*race

^3^ *p* < 0.10 (each group vs. reference group). Reference group defined as no PCOS, HA, or OA. Baseline group = White. Black sample sizes: PCOS *N* = 11, OA *N* = 26, Reference *N* = 256; race-specific results are derived from the interaction model. White sample sizes: PCOS *N* = 29, OA *N* = 49, Reference *N* = 273; race-specific results are derived from the interaction model

## Discussion

This study investigated the associations of diet and physical activity with PCOS, an important topic given the use of health-related behavioral modifications to treat and prevent PCOS [38]. Cross-sectional analyses in the CARDIA cohort revealed total daily energy, nutrient intake, diet quality, and physical activity had no association with the odds of PCOS and isolated features. Further analyses also showed a potential impact of race, albeit these findings were from a small sample of women with PCOS.

There were no differences in macro- and micronutrient intakes between women with and without PCOS in the CARDIA cohort. Previous U.S. [14, 17] and international studies [15, 16, 18] mainly reported no differences in total energy and/or macro- or micronutrient intake between women with and without PCOS, although three prior studies found that women with PCOS consumed more carbohydrates (ranged from 31 to 43 g) than comparison groups [9, 10, 14]. The conflicting evidence may be explained by the use of unexplained or unique criteria to define PCOS (i.e., luteinizing hormone: follicle stimulating hormone) in these earlier studies, and/or their comparison of women with PCOS to infertile women with various etiologies (rather than a comparison to reproductively healthy women). Our study of relatively younger women, taken together with another US study of older women [14], suggests that there are no differences in total daily energy intake between women with and without PCOS during the reproductive and peri-menopausal years. Additionally, when examining diet quality of women with PCOS in the US, our finding that there were no differences in diet quality between women with and without PCOS confirmed our observations from a recent study with a different US participant sample [39].

Our findings of no associations between nutrients with OA contrast with two longitudinal cohort studies that reported a greater intake of carbohydrate and folic acid was associated with a reduced risk of anovulatory infertility [40, 41] but agree with findings of a longitudinal cohort study of B vitamins and self-reported anovulatory infertility, which reported no significant associations [41]. The inconsistencies between study findings may be attributed to the heterogeneity in the causes of HA and OA [42]. For example, anovulatory infertility can manifest secondary to stress and nutrient deficiency, and not all women with OA are at risk of progressing to PCOS due to differences in etiology [43]. We did not observe any associations of diet and physical activity with HA.

There was little to no association of physical activity behaviors with PCOS, which agrees with previous studies that noted no differences in self-reported moderate and vigorous intensity physical activity between women with and without PCOS [8]. Similar to previous studies that investigated physical activity in adult women with PCOS [11–14, 18], our findings do not support the hypothesis that women with PCOS engage in less moderate and vigorous physical activity [38]. Other researchers reported differences in sedentary and physical activity patterns in women with PCOS (i.e. longer sitting intervals), which have been associated with increased risk of all-cause mortality [44]. The CARDIA measurement of physical activity did not capture patterns of exercise types and sedentary behaviors across time, and thus, objective physical activity data are needed to assess the full spectrum of movement intensity, from sedentary to vigorous activities.

Given that racial differences in the risk of glucoregulatory and cardiometabolic conditions have been identified in PCOS patients [23–26], a modifying effect of race on the associations between diet and physical activity with PCOS warranted consideration. We found that a higher whole grain intake, and a lower sugar-sweetened beverage and fruit juice intake, were associated with higher odds of PCOS and/or OA in Black women. These findings are paradoxical given that these two dietary patterns are typically associated with better health outcomes [45]. Our conclusions are limited by the small number of Black women with PCOS in this study and the multiple tests for interaction that may lead to type I errors.

Strengths of this study include the lower likelihood of reverse causality as an explanation for these findings because diet and physical activity data were collected prior to widespread recognition of PCOS, which mainly occurred after the establishment of formal NIH (1990) criteria for PCOS. The cohort also contained an equal proportion of Black and White participants, which provided the opportunity to explore differential diet, physical activity and PCOS associations by race. Notably, the CARDIA Women’s study was developed to identify those with PCOS features within the CARDIA cohort, thus allowing us and others to evaluate the isolated and combined features of PCOS using a well-established dataset [31, 46–48]. A limitation of this study includes the potential residual confounding by AHEI-2010 groups that are highly correlated, although there will be minimal issue with groups that are not closely associated with one another. Additionally, there is greater potential for type I error due to multiple comparisons, therefore our study results should be considered exploratory. Further, self-reported and retrospective accounts of menstrual cycle history and clinical signs of androgen excess were not confirmed with clinical assessments. Our findings should also be viewed as being relevant for the most severe clinical phenotype of PCOS (defined as hyperandrogenism and irregular menses) because the absence of data on ovarian morphology meant we could not consider the association of diet and/or physical activity with the other phenotypes of PCOS. Future studies of how diet and physical activity associate with outcomes across the various PCOS definitions are needed particularly in light of the recent release of international evidence-based guidelines for PCOS, which support ovarian morphology as a PCOS criterion [29]. Previous studies have identified nutrient predictors of anovulatory infertility [40, 41], but associations between food groups and physical activity with PCOS remained largely unexplored. Though nutrients are important components of the diet, evaluation of diet quality - particularly by race - provides further knowledge about the potential targets for intervention since alterations in foods and/or dietary patterns, rather than nutrients, are critical information for patients.

## Conclusions

Overall, results from this study revealed no differences in diet and physical activity between those with and without isolated or combined features of PCOS. This study found preliminary evidence that associations between food groups and PCOS may differ by race. Findings from this study are formative and add to the current literature to promote greater understanding about the association of diet and physical activity on the development of PCOS.

## Supplementary Information

**Additional file 1.**

## Data Availability

The data that support the findings of this study are available from the CARDIA field centers but restrictions apply to the availability of these data, which were used under license for the current study, and so are not publicly available. The authors obtained permission from the CARDIA Coordinating Center to access the data needed to meet the study objectives.

## Abbreviations

AHEI-2010: Alternate Healthy Eating Index 2010
BMI: Body Mass Index
CARDIA: Coronary Artery Risk Development in Young Adults
CWS: CARDIA Women’s Study
EPA + DHA: Eicosapentaenoic and Docosahexaenoic Acids
EU: Exercise Units
HA: Isolated Hyperandrogenism (elevated testosterone and/or hirsutism at 2 sites or more)
IU: International Units; Reference (neither PCOS nor HA nor OA)
MUFA: Monounsaturated Fatty Acid
NIH: National Institutes of Health
NDSR: Nutrient Data Software for Research
ng/dL: Nanograms per Deciliter
OA: Isolated Oligomenorrhea (≥34 days in menstrual cycle)
PCOS: Polycystic Ovary Syndrome (hyperandrogenism and oligomenorrhea)
PA: Physical Activity
PUFA: Polyunsaturated Fatty Acid
SD: Standard Deviation
