# Supplementary material for: Associations of diet, physical activity and polycystic ovary syndrome in the Coronary Artery Risk Development in Young Adults Women’s Study
Source: BMC Public Health. 2021 Jan 6;21:35. doi: 10.1186/s12889-020-10028-5 (PMC7789704; doi:10.1186/s12889-020-10028-5)
Supplement: Supplementary file 1 — Additional file 1. [file 12889_2020_10028_MOESM1_ESM.docx]

**Online Supplementary Material**


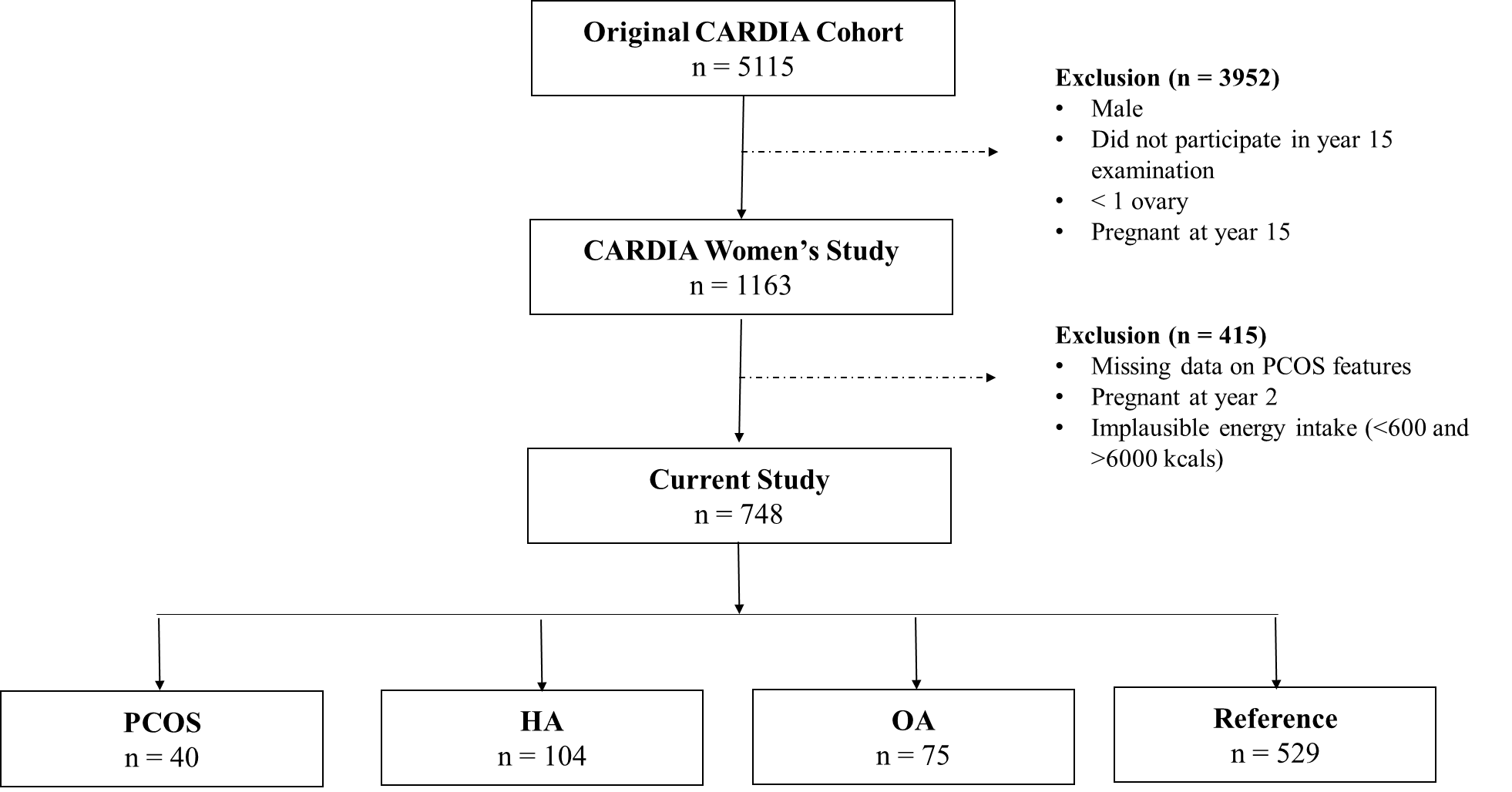


**Supplemental Figure 1: Participant Flowchart.** *Abbreviations: Coronary Artery Risk Development in Young Adults, CARDIA; HA, hyperandrogenism; kcal, Calories; OA, oligomenorrhea; polycystic ovary syndrome.*

| Supplemental Table 1. Polytomous logistic regression models estimating the associations of nutrients and physical activity with odds of reproductive status^1^ | | | | |
| --- | --- | --- | --- | --- |
| **Variables** | **Partial Model** | | **Full Model** | |
|  | **Odds Ratio** | **95% CI** | **Odds Ratio** | **95% CI** |
| Total Carbohydrate (g/d) |  |  |  |  |
| PCOS | 1.00 | 0.99, 1.01 | 1.00 | 0.99, 1.01 |
| HA | 1.00 | 1.00, 1.01 | 1.00 | 1.00, 1.01 |
| OA | 1.00 | 1.00, 1.01 | 1.00 | 1.00, 1.01 |
| Fiber (g/d) |  |  |  |  |
| PCOS | 0.92 | 0.79, 1.07 | 0.94 | 0.80, 1.09 |
| HA | 1.04 | 0.95, 1.14 | 1.04 | 0.95, 1.14 |
| OA | 1.03 | 0.93, 1.15 | 1.04 | 0.93, 1.15 |
| Total Protein (g/d) |  |  |  |  |
| PCOS | 1.00 | 0.98, 1.02 | 1.00 | 0.98, 1.02 |
| HA | 1.00 | 0.99, 1.01 | 1.00 | 0.99, 1.01 |
| OA | 0.99 | 0.97, 1.01 | 0.99 | 0.97, 1.01 |
| Total Fat (g/d) |  |  |  |  |
| PCOS | 1.01 | 0.98, 1.03 | 1.01 | 0.99, 1.03 |
| HA | 1.00 | 0.99, 1.01 | 1.00 | 0.99, 1.01 |
| OA | 1.00 | 0.99, 1.02 | 1.00 | 0.99, 1.02 |
| Cholesterol (mg/d) |  |  |  |  |
| PCOS | 1.00 | 1.00, 1.00 | 1.00 | 1.00, 1.00 |
| HA | 1.00 | 1.00, 1.00 | 1.00 | 1.00, 1.00 |
| OA | 1.00 | 1.00, 1.00 | 1.00 | 1.00, 1.00 |
| Total SFA (g/d) |  |  |  |  |
| PCOS | 1.01 | 0.96, 1.05 | 1.01 | 0.97, 1.05 |
| HA | 0.98 | 0.96, 1.01 | 0.98 | 0.96, 1.01 |
| OA | 0.99 | 0.96, 1.02 | 0.99 | 0.96, 1.03 |
| Total MUFA (g/d) |  |  |  |  |
| PCOS | 1.03 | 0.98, 1.08 | 1.03 | 0.98, 1.08 |
| HA | 1.00 | 0.97, 1.03 | 1.00 | 0.97, 1.03 |
| OA | 1.00 | 0.96, 1.03 | 1.00 | 0.96, 1.03 |
| Total PUFA (g/d) |  |  |  |  |
| PCOS | 0.98 | 0.92, 1.05 | 0.98 | 0.92, 1.05 |
| HA | 1.02 | 0.98, 1.05 | 1.02 | 0.98, 1.05 |
| OA | 1.03 | 0.99, 1.07 | 1.03 | 0.99, 1.07 |
| Omega 3 (mg/day) |  |  |  |  |
| PCOS | 1.00 | 1.00, 1.00 | 1.00 | 1.00, 1.00 |
| HA | 1.00 | 1.00, 1.00 | 1.00 | 1.00, 1.00 |
| OA | 1.00 | 1.00, 1.00 | 1.00 | 1.00, 1.00 |
| Vitamin A (IU/d) |  |  |  |  |
| PCOS | 1.00 | 1.00, 1.00 | 1.00 | 1.00, 1.00 |
| HA | 1.00 | 1.00, 1.00 | 1.00 | 1.00, 1.00 |
| OA | 1.00 | 1.00, 1.00 | 1.00 | 1.00, 1.00 |
| Vitamin C (mg/d) |  |  |  |  |
| PCOS | 1.00 | 1.00, 1.00 | 1.00 | 1.00, 1.00 |
| HA | 1.00 | 1.00, 1.00 | 1.00 | 1.00, 1.00 |
| OA | 1.00 | 1.00, 1.00 | 1.00 | 1.00, 1.00 |
| Vitamin D (mcg /d) |  |  |  |  |
| PCOS | 0.97 | 0.91, 1.04 | 0.98 | 0.91, 1.04 |
| HA | 1.02 | 0.99, 1.06 | 1.03 | 1.00, 1.06 |
| OA | 0.98 | 0.94, 1.03 | 0.98 | 0.94, 1.03 |
| Alpha Tocopherol Equivalents (mg/d) | | | | |
| PCOS | 1.00 | 1.00, 1.01 | 1.00 | 1.00, 1.01 |
| HA | 1.00 | 1.00, 1.01 | 1.00 | 1.00, 1.01 |
| OA | 0.99 | 0.97, 1.01 | 0.99 | 0.97, 1.01 |
| Sodium (mg/d) |  |  |  |  |
| PCOS | 1.00 | 1.00, 1.00 | 1.00 | 1.00, 1.00 |
| HA | 1.00 | 1.00, 1.00 | 1.00 | 1.00, 1.00 |
| OA | 1.00 | 1.00, 1.00 | 1.00 | 1.00, 1.00 |
| Calcium (mg/d) |  |  |  |  |
| PCOS | 1.00 | 1.00, 1.00 | 1.00 | 1.00,1.00 |
| HA | 1.00 | 1.00, 1.00 | 1.00 | 1.00, 1.00 |
| OA | 1.00 | 1.00, 1.00 | 1.00 | 1.00, 1.00 |
| Phosphorus (mg/d) |  |  |  |  |
| PCOS | 1.00 | 1.00, 1.00 | 1.00 | 1.00, 1.00 |
| HA | 1.00 | 1.00, 1.00 | 1.00 | 1.00, 1.00 |
| OA | 1.00 | 1.00, 1.00 | 1.00 | 1.00, 1.00 |
| Thiamin (mg/d) |  |  |  |  |
| PCOS | 0.82 | 0.60, 1.13 | 0.83 | 0.60, 1.15 |
| HA | 1.06 | 0.98, 1.14 | 1.06 | 0.99, 1.14 |
| OA | 0.86 | 0.70, 1.06 | 0.87 | 0.70, 1.07 |
| Potassium (mg/d) |  |  |  |  |
| PCOS | 1.00 | 1.00, 1.00 | 1.00 | 1.00, 1.00 |
| HA | 1.00 | 1.00, 1.00 | 1.00 | 1.00, 1.00 |
| OA | 1.00 | 1.00, 1.00 | 1.00 | 1.00, 1.00 |
| Riboflavin (mg/d) |  |  |  |  |
| PCOS | 0.87 | 0.67, 1.12 | 0.88 | 0.68, 1.13 |
| HA | 1.05 | 0.97, 1.13 | 1.05 | 0.98, 1.13 |
| OA | 0.87 | 0.72, 1.05 | 0.87 | 0.72, 1.05 |
| Niacin (mg/d) |  |  |  |  |
| PCOS | 0.98 | 0.96, 1.01 | 0.98 | 0.96, 1.01 |
| HA | 1.01 | 1.00, 1.01 | 1.01 | 1.00, 1.01 |
| OA | 0.99 | 0.97, 1.01 | 0.99 | 0.97, 1.01 |
| Iron (mg/d) |  |  |  |  |
| PCOS | 0.99 | 0.97, 1.02 | 1.00 | 0.98, 1.02 |
| HA | 1.00 | 0.99, 1.01 | 1.00 | 0.99, 1.01 |
| OA | 0.99 | 0.98, 1.01 | 0.99 | 0.98, 1.01 |
| Copper (mg/d) |  |  |  |  |
| PCOS | 0.85 | 0.64, 1.14 | 0.86 | 0.64, 1.15 |
| HA | 1.07 | 0.96, 1.20 | 1.08 | 0.96, 1.21 |
| OA | 1.04 | 0.91, 1.20 | 1.05 | 0.91, 1.21 |
| Magnesium (mg/d) |  |  |  |  |
| PCOS | 1.00 | 1.00, 1.00 | 1.00 | 1.00, 1.00 |
| HA | 1.00 | 1.00, 1.00 | 1.00 | 1.00, 1.00 |
| OA | 1.00 | 1.00, 1.00 | 1.00 | 1.00, 1.00 |
| Zinc (mg/d) |  |  |  |  |
| PCOS | 0.96 | 0.91, 1.02 | 0.96 | 0.91, 1.02 |
| HA | 1.01 | 1.00, 1.03 | 1.02 | 1.00, 1.03 |
| OA | 1.00 | 0.98, 1.03 | 1.00 | 0.98, 1.03 |
| Folic Acid (mcg/d) |  |  |  |  |
| PCOS | 1.00 | 1.00, 1.00 | 1.00 | 1.00, 1.00 |
| HA | 1.00 | 1.00, 1.00 | 1.00 | 1.00, 1.00 |
| OA | 1.00 | 1.00, 1.00 | 1.00 | 1.00, 1.00 |
| Caffeine (mg/d) |  |  |  |  |
| PCOS | 1.00 | 1.00, 1.00 | 1.00 | 1.00, 1.00 |
| HA | 1.00 | 1.00, 1.00 | 1.00 | 1.00, 1.00 |
| OA | 1.00 | 1.00, 1.00 | 1.00 | 1.00, 1.00 |
| **Physical Activity** | | | | |
| Moderate Exercise |  |  |  |  |
| PCOS | 1.00 | 1.00, 1.00 | 1.00 | 1.00, 1.00 |
| HA | 1.00 | 1.00, 1.00 | 1.00 | 1.00, 1.00 |
| OA | 1.00 | 1.00, 1.00 | 1.00 | 1.00, 1.00 |
| Heavy Exercise |  |  |  |  |
| PCOS | 1.00 | 1.00, 1.00 | 1.00 | 1.00, 1.00 |
| HA | 1.00 | 1.00, 1.00 | 1.00 | 1.00, 1.00 |
| OA | 1.00 | 1.00, 1.00 | 1.00 | 1.00, 1.00 |
| Total Exercise |  |  |  |  |
| PCOS | 1.00 | 1.00, 1.00 | 1.00 | 1.00, 1.00 |
| HA | 1.00 | 1.00, 1.00 | 1.00 | 1.00, 1.00 |
| OA | 1.00 | 1.00, 1.00 | 1.00 | 1.00, 1.00 |
| ^1^ HA, Isolated Hyperandrogenism (elevated testosterone and/or hirsutism at 2 sites or more); IU, International Units; MUFA, Monounsaturated Fatty Acid; OA, Isolated Oligomenorrhea (≥34 days in menstrual cycle); PCOS. Polycystic Ovary Syndrome (hyperandrogenism and oligomenorrhea); PUFA, Polyunsaturated Fatty Acid; SFA, Saturated Fatty Acid  ^2^ Partially adjusted model adjusted for covariates: age, race, total energy intake, education. Fully adjusted model adjusted for covariates in partial model plus body mass index.  ^3^Overall significance level P<0.05 (each group vs. reference group). Reference group defined as no PCOS, HA, or OA. | | | | |

| Supplemental Table 2. Polytomous logistic regression models estimating the associations of diet quality scores with odds of reproductive status^1^ | | | | |
| --- | --- | --- | --- | --- |
| **AHEI-2010^a^ Subcomponent Scores** | **Partial Model** | | **Full Model** | |
|  | **Odds Ratio** | **95% CI** | **Odds Ratio** | **95% CI** |
| **Vegetables** | | | | |
| PCOS | 0.94 | 0.82, 1.08 | 0.95 | 0.83, 1.09 |
| HA | 1.01 | 0.93, 1.11 | 1.02 | 0.93, 1.11 |
| OA | 1.03 | 0.93, 1.13 | 1.03 | 0.93, 1.14 |
| **Fruits** | | | | |
| PCOS | 0.96 | 0.84, 1.10 | 0.97 | 0.85, 1.12 |
| HA | 1.04 | 0.96, 1.14 | 1.05 | 0.97, 1.14 |
| OA | 1.00 | 0.91, 1.11 | 1.01 | 0.91, 1.11 |
| **Whole Grains** | | | | |
| PCOS | 0.98 | 0.84, 1.14 | 0.99 | 0.85, 1.16 |
| HA | 0.93 | 0.84, 1.04 | 0.94 | 0.85, 1.05 |
| OA | 0.94 | 0.83, 1.06 | 0.95 | 0.84, 1.07 |
| **Sugar Sweetened Beverages, Fruit Juice** | | | | |
| PCOS | 1.04 | 0.93, 1.17 | 1.05 | 0.94, 1.18 |
| HA | 1.03 | 0.95, 1.12 | 1.03 | 0.95,1.12 |
| OA | 1.01 | 0.92, 1.10 | 1.01 | 0.93, 1.10 |
| **Nuts and Legumes** | | | | |
| PCOS | 1.00 | 0.91, 1.11 | 1.00 | 0.91, 1.11 |
| HA | 0.98 | 0.92, 1.05 | 0.98 | 0.91, 1.05 |
| OA | 0.95 | 0.88, 1.03 | 0.95 | 0.88, 1.03 |
| **Red, Processed Meats** | | | | |
| PCOS | 0.90 | 0.80, 1.01 | 0.92 | 0.81, 1.03 |
| HA | 1.00 | 0.92, 1.08 | 1.01 | 0.93, 1.10 |
| OA | 0.99 | 0.90, 1.08 | 1.00 | 0.91, 1.09 |
| ***trans*-Fat** | | | | |
| PCOS | 0.91 | 0.67, 1.24 | 0.88 | 0.65, 1.21 |
| HA | 1.00 | 0.82, 1.21 | 0.98 | 0.81, 1.20 |
| OA | 0.93 | 0.74, 1.17 | 0.92 | 0.73, 1.15 |
| **Long Chain Omega 3 Fats** | | | | |
| PCOS | 1.00 | 0.88, 1.13 | 1.01 | 0.89, 1.15 |
| HA | 1.05 | 0.97, 1.13 | 1.06 | 0.98, 1.14 |
| OA | 1.05 | 0.96, 1.15 | 1.05 | 0.96, 1.16 |
| **PUFA** | | | | |
| PCOS | 0.99 | 0.84, 1.16 | 1.00 | 0.85, 1.17 |
| HA | 1.07 | 0.96, 1.18 | 1.07 | 0.97, 1.19 |
| OA | 1.05 | 0.94, 1.19 | 1.05 | 0.94, 1.19 |
| **Sodium** | | | | |
| PCOS | 0.94 | 0.77, 1.15 | 0.95 | 0.77, 1.17 |
| HA | 0.99 | 0.87, 1.12 | 0.99 | 0.87, 1.13 |
| OA | 0.97 | 0.84, 1.13 | 0.98 | 0.84, 1.13 |
| **Alcohol** | | | | |
| PCOS | 1.03 | 0.91, 1.15 | 1.05 | 0.93, 1.18 |
| HA | 1.06 | 0.99, 1.14 | 1.07 | 0.99, 1.16 |
| OA | 0.98 | 0.90, 1.06 | 0.98 | 0.90, 1.07 |
| **Total AHEI-2010 Score** | | | | |
| PCOS | 0.99 | 0.96, 1.02 | 1.00 | 0.97, 1.03 |
| HA | 1.01 | 0.99, 1.03 | 1.01 | 0.99, 1.03 |
| OA | 1.00 | 0.97, 1.02 | 1.00 | 0.98, 1.02 |

^1^ AHEI-2010, Alternative Healthy Eating Index 2010; HA, Isolated Hyperandrogenism (elevated testosterone and/or hirsutism at 2 sites or more); OA, Isolated Oligomenorrhea (≥34 days in menstrual cycle); Reference (neither PCOS nor HA nor OA); PCOS, Polycystic Ovary Syndrome (hyperandrogenism and oligomenorrhea); PUFA, Polyunsaturated Fatty Acid; SFA, Saturated Fatty Acid; SSB, Sugar-sweetened Beverages

^2^ Partially adjusted model adjusted for covariates: age, race, total energy intake, education. Fully adjusted model adjusted for covariates in partial model plus body mass index.

^3^ P<0.05 (each group vs. reference group). Reference group defined as no PCOS, HA, or OA.
